# Shallow V-Shape Nanostructured Pit Arrays in Germanium Using Aqua Regia Electroless Chemical Etching

**DOI:** 10.3390/ma10080854

**Published:** 2017-07-26

**Authors:** Ibtihel Chaabane, Debika Banerjee, Oualid Touayar, Sylvain G. Cloutier

**Affiliations:** 1 Department of Electrical Engineering, École de Technologie Supérieure, 1100 Notre-Dame Ouest, Montréal, Québec, QC H3C 1K3, Canada; ibtihel.chaabane.1@etsmtl.net (I.C.); debika.banerjee.1@etsmtl.net (D.B.); 2 Department of Physics and Instrumentation, National Institute of Applied Science and Technologies, Charguia, Tunis 1080, Tunisia; touayar.walid@planet.tn

**Keywords:** Germanium, wet electroless etching, aqua regia, nanostructures, optical properties

## Abstract

Due to its high refractive index, reflectance is often a problem when using Germanium for optoelectronic devices integration. In this work, we propose an effective and low-cost nano-texturing method for considerably reducing the reflectance of bulk Germanium. To do so, uniform V-shape pit arrays are produced by wet electroless chemical etching in a 3:1 volume ratio of highly-concentrated hydrochloridric and nitric acids or so-called aqua regia bath using immersion times ranging from 5 to 60 min. The resulting pit morphology, the crystalline structure of the surface and the changes in surface chemistry after nano-patterning are all investigated. Finally, broadband near-infrared reflectance measurements confirm a significant reduction using this simple wet etching protocol, while maintaining a crystalline, dioxide-free, and hydrogen-passivated surface. It is important to mention that reflectance could be further reduced using deeper pits. However, most optoelectronic applications such as photodetectors and solar cells require relatively shallow patterning of the Germanium to allow formation of a pn-junction close to the surface.

## 1. Introduction

The instability of Germanium dioxide (GeO_2_) was a primary cause for the abandonment of Germanium (Ge) in favor of Silicon as the premier material platform for microelectronics [1,2]. Nevertheless, Ge still offers many unique properties for optoelectronic devices [3,4,5,6,7,8], especially for near-infrared applications [9].

However, a major inconvenience with bulk Ge is its high refractive index, which causes large reflections, preventing efficient light coupling. In the last two decades, nanoscale texturing has proved to be a successful approach to reduce the broadband reflectance of semiconductor surfaces [10,11,12,13,14]. While many low-cost nano-texturing processes were pioneered for silicon [15,16,17,18,19], researchers have been searching for an equivalent approach to achieve uniform and low-cost anti-reflection for bulk Germanium. Some earlier reports focused on electrochemical etching [20,21,22,23,24], where electric fields are used to drive the reaction. Different types of etching agents for various concentrations and etching times were used and encouraging results were achieved using this process [20]. To allow for large-scale manufacturing at low costs, it would be better if an electroless approach could achieve similar results.

Since no good electroless recipe was found, we went back to the roots of chemical wet etching for microelectronics. With the push to develop microelectronics, researchers quickly sought low-cost chemical etching processes to achieve uniform cleaning and etching [2,20,22,25,26,27,28,29,30,31,32]. Early works quickly converged around two main standard cleaning (SC) protocols pioneered in the late 1960s and early 1970s [25,33].

In the last decade, some research groups have attempted to adapt these standard protocols for Germanium. Regarding HCl-based cleaning protocols, the transfer of aqueous-based cleaning (H_2_O:H_2_O_2_:HCl) from Silicon to Germanium was first attempted as early as 1970, but reports concluded that the protocols should be adjusted to correct for the solubility of GeO_2_ in water [34]. More recently, reports looking at the effectiveness of using HCl instead of HF to remove oxide and metal contamination revealed that repetitive treatments with high-concentration HCl is a good protocol for Ge cleaning [34]. Meanwhile, a broader comparison between different acids including HF, HCl, HBr, and HI as cleaning agents proposed HBr and HI as the best cleaning agents for Ge [34]. However, a similar study also concluded that HCl is more effective to remove oxides and improve surface smoothness for Ge [34].

Simultaneously, researchers also explored the use of a mixture of H_2_O_2_:base (KOH, NH_3_) as an etching agent for Germanium. This approach directly derives from the SC protocol for Silicon using H_2_O:H_2_O_2_:NH_3_ [25], where the H_2_O_2_ acts as an oxidizing agent [34]. Other groups also explored the use of H_2_O_2_ as an oxidizing agent, but using an acid (HCl) instead of a base for the solution [34]. They studied the dissolution kinetics of Germanium, comparing the results of electroless wet etching using HCl:H_2_O_2_ to electrochemical etching using HCl. However, the electroless results show an important non-uniformities in the roughness depending on the HCl concentration, and demonstrate that HCl electrochemical etching leads to the formation of tall pyramids of about 4 µm in height [34], a major problem when shallow pn junction devices are needed.

In this work, we report a new electroless chemical etching treatment to achieve uniform and shallow Ge nanopatterning yielding reduced optical reflectance for optoelectronic applications. This protocol relies on a specific mixture of HCl:HNO_3_ at 3:1 concentrations or so-called aqua regia. Our analysis relies on SEM and AFM analysis to study the film morphology and surface characteristics. In addition, we use XRD, FTIR, and XPS to assess crystallinity and confirm a hydrogen passivation of the treated surface, which protects from native oxidation when exposed to air. Finally, reflectance measurements confirm that this electroless all solution-based protocol can significantly reduce reflectance using shallow nano-texturing of Germanium for device integration.

## 2. Results and Discussion

### 2.1. Structural Characterization by SEM

**Figure 1:**
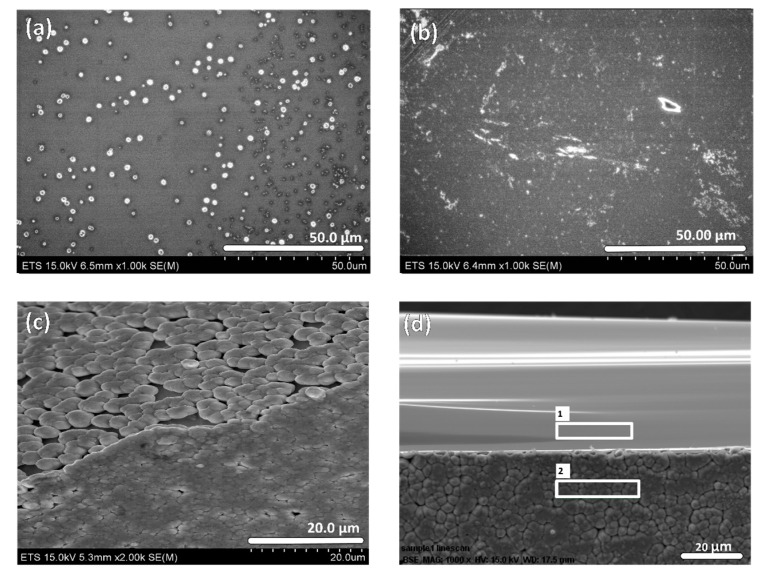
(**a**) Pristine n-type and Sb-doped Ge (100) surface without cleaning; (**b**) Sample after a 15-min immersion in concentrated 37% HCl bath; (**c**) Tilted (45°) view of a Ge sample’s edge observed after a 60-min immersion in 70% HNO_3_ bath; (**d**) Cross-sectional view of a Ge sample cleaved after a 60-min immersion in 70% HNO_3_ bath. Section 1 and Section 2 were analyzed by EDX and their compositions are summarized in Table 1.

While Figure 1a represents a typical Germanium sample (as received), Figure 1b shows the surface after cleaning in a 37% HCl bath for 15 min at room temperature to remove the impurities observed on the cleaved wafers. Then, immersion in a 70% HNO_3_ bath leads to a pronounced surface oxidation. Indeed, the SEM image shown in Figure 1c is a 45°-tilted top view of the edge of a clean Ge sample after 60 min oxidation in 70% HNO_3_. Finally, the sample shown in Figure 1c was cleaved again using a diamond scriber to reveal a pristine edge used as a baseline to identify the species formed during HNO_3_ bath immersion. Figure 1d shows the cleaved cross-section, identifying the two distinct regions analyzed under EDX with their respective elemental compositions shown in Table 1. Section 1 is the fresh untreated Ge and Section 2 is the oxidized GeO*_x_* layer formed during HNO_3_ bath immersion.

When using a mixture of HCl:HNO_3_, we are expecting that HNO_3_ will act as an oxidizing agent for Ge and will also bump HCl molecules off till their separation to let them further dissolve the Germanium. Hence, HCl (a halide like HF, HBr, and HI) will help to weaken interatomic bonds, allowing etching. As such, the experimental plan for such a mixture involves three key parameters: (1) HCl concentration; (2) HNO_3_ concentration; and (3) bath temperature and immersion time. Here, we settled for a 3:1 solution of HCl:HNO_3_ (aqua regia), and all immersions were performed at room temperature. We choose this particular aqua regia volumetric mixture 3:1 of concentrated HCl and HNO_3_ because this makes the solution stronger which will attack more aggressively the Germanium substrate. 4:1 or 5:1 has less HNO_3_ available so it is less aggressive.

**Figure 2:**
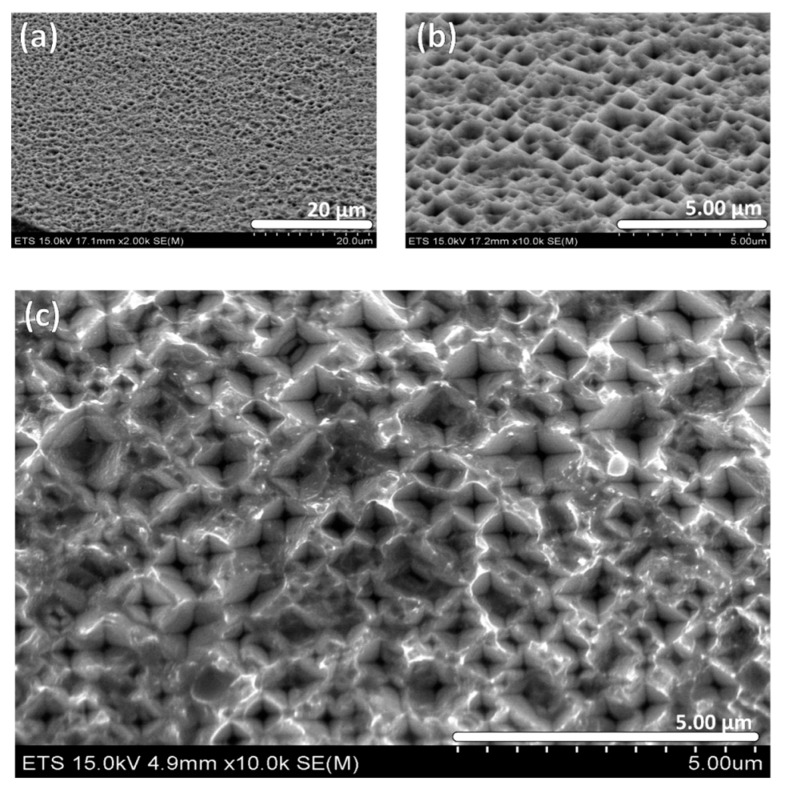
Etched Germanium sample after 15-min treatment in 3:1 Aqua Regia. (**a**) Tilted (45°) view at low resolution; (**b**) Tilted (45°) view at higher resolution; (**c**) Top-view of the inverted-pyramid pit structure at high resolution.

Figure 2 shows typical SEM micrographs after immersion in the 3:1 aqua regia bath for 15 min at room temperature. The low-resolution micrograph in Figure 2a confirms the surface is uniformly etched, while the higher-resolution micrographs in Figure 2b,c clearly show the inverted-pyramid pit structures, indicating the anisotropy of the chemical etching process. This formation could be explained by the etching that happens along the low-energy planes of the diamond crystalline structure of the Ge.

**Figure 3:**
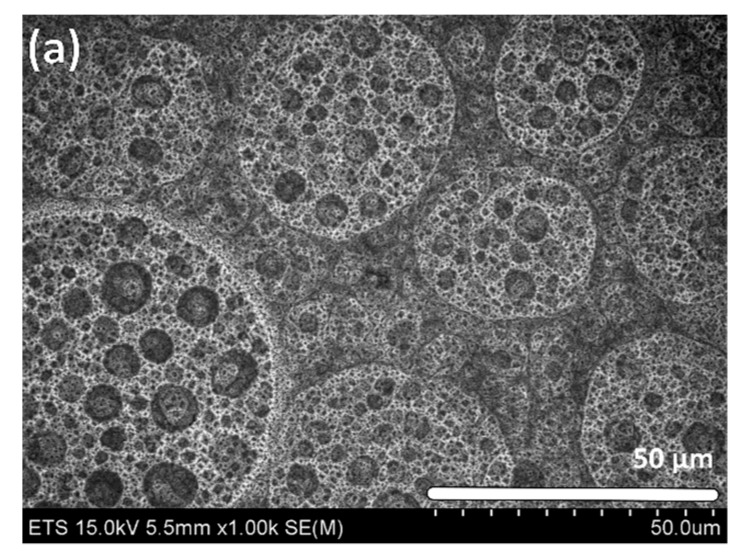

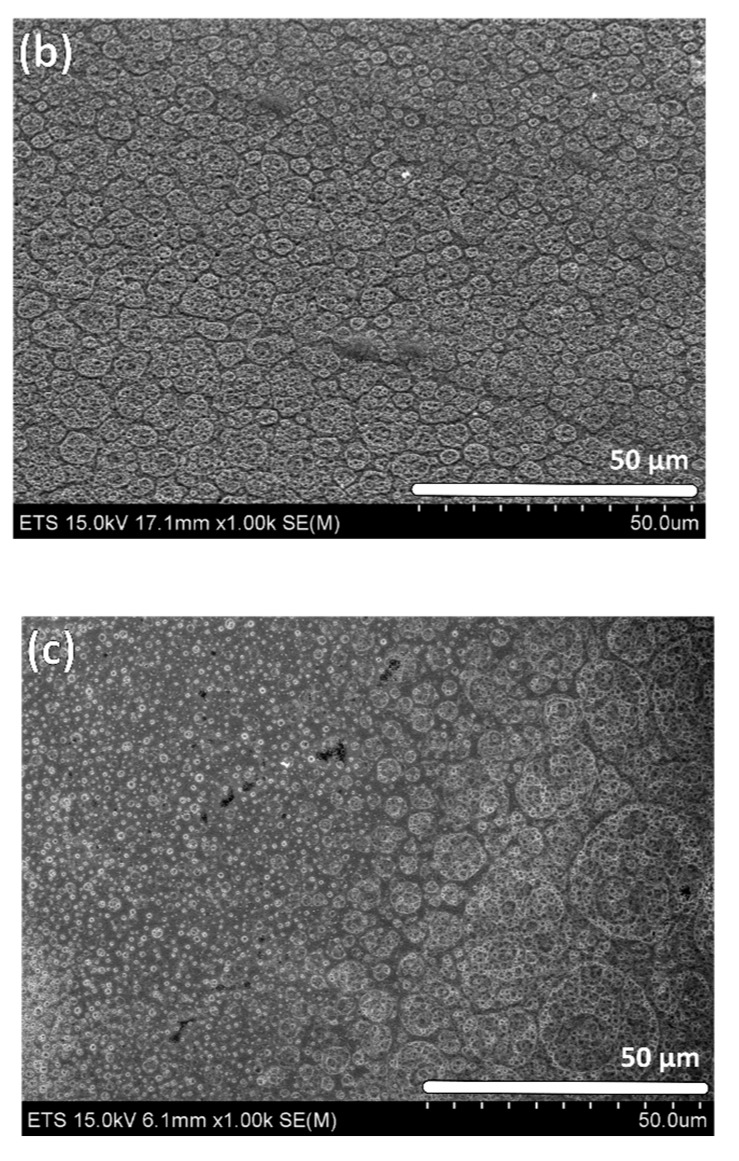
Effect of the wetting agent (ethanol) on the H_2_-bubbling consequences. (**a**) Top-view of a Ge sample following a 7-min immersion in aqua regia; (**b**) Tilted (45°) view of a Ge sample following a 7-min immersion in a mix of aqua regia with ethanol; (**c**) Top-view of (b).

Lower magnification images illustrated in Figure 3 show the formation of long-range second-order circle-like features in the etching pattern, which are more clearly observed for very short (7-min) immersion times. These features stem from a H_2_-bubbling phenomenon [35], which was later confirmed by chemical analysis of the surface. Stirring or depositing the beaker in the top of a vortex shaker in order to release the surface from the bubbles doesn’t show any improvement: Neither by bare eyes nor under SEM microscope. However, a possible solution to alleviate this H_2_-bubbling effect from the surface patterning will be to add a wetting agent to the solution.

To validate this hypothesis, we added ethanol in the same proportion as HNO_3_ as a wetting agent in the solution. While the long-range circle-like features are certainly much less pronounced in Figure 3b, we find that some remnants of residual H_2_-bubbling can still be seen on the wafer surface as shown in Figure 3c. Also, the reaction is not stable after about 7 min due to the probable formation of ethyl nitrate, CH_3_CH_2_ONO_2_ [36]. So this enhancement is possible only for very short immersion times. However, this is not an issue here, as these features blend-in as the surface corrugations increases for longer etching times and the H_2_ bubbles, when formed, are immediately released due to the surface passivation that will be discussed below.

### 2.2. Surface Morphology Studied by AFM

**Figure 4:**
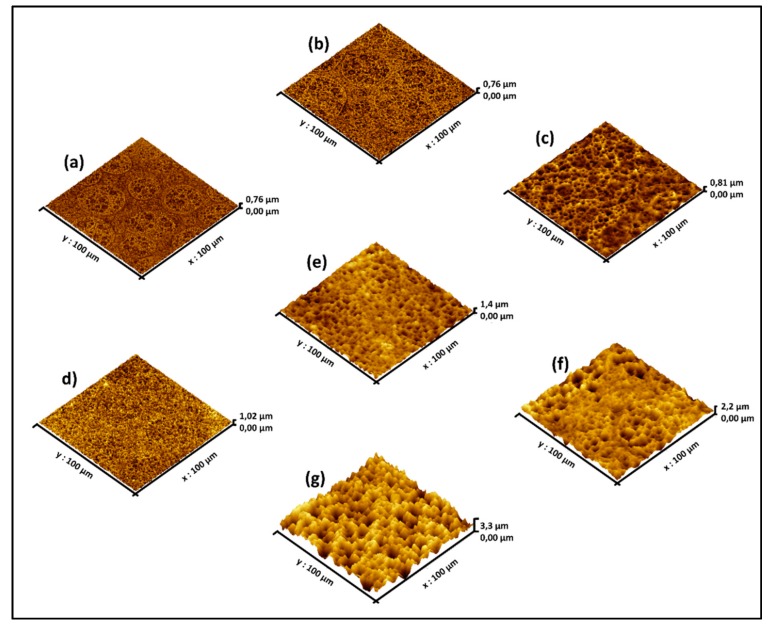
AFM images of Ge samples immersed in Aqua regia for (**a**) 5 min; (**b**) 7 min; (**c**) 10 min; (**d**) 12 min; (**e**) 15 min; (**f**) 30 min; and (**g**) 60 min.

More careful AFM analysis shown in Figure 4 indicates how the surface morphology evolves by increasing the immersion time from 5 to 60 min. Except for some residual H_2_ bubbling marks discussed in the previous section, the porous pit structures have distributed uniformly on the surface. Table 2 compares the surface statistics including roughness (*R_a_*), mean and maximum heights (relative on the lowest point taken as reference) for increasing immersion times.

**Figure 5:**
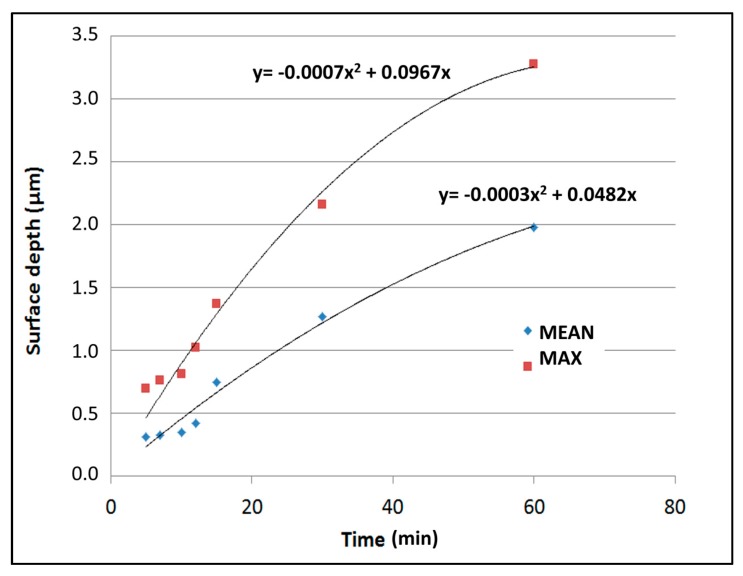
Evolution of the nanostructured surface depth with increasing immersion time in the aqua regia bath.

Clearly, longer immersion times lead to a deeper etching. However, H_2_ bubbling that stays attached to the surface eventually causes the etching to slow down in some regions and some stirring inside the solution. In Figure 5, we can observe a significant slowdown of the etching rate for longer immersion. We have explained this phenomenon in the next sections, when delving in to the surface chemistry analysis.

### 2.3. Structural Analysis Using X-ray Diffraction

**Figure 6:**
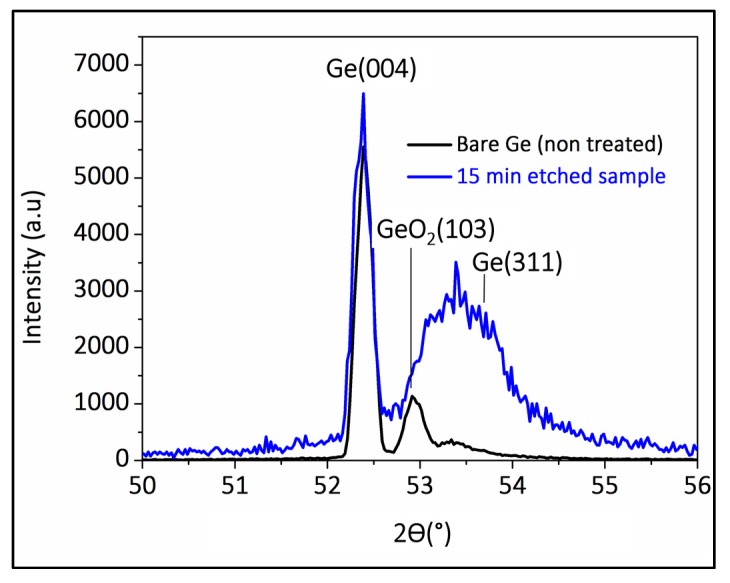
X-ray diffraction (XRD) of the cleaned Germanium before and after a 15-min immersion in the aqua regia solution.

Surface analysis using X-ray diffraction (XRD) has performed to determine the crystallinity of the etched sample and the crystallite size. The XRD data from Figure 6 reveals peaks corresponding to three different Ge lattice planes [37,38].

The main peak corresponds to the (004) crystallographic orientation, followed by a second less intense peak closer to 53° and corresponding to the (103) crystallographic orientation for the bare Germanium. After etching, a broader peak centered at 53.5° corresponding to the (311) crystallographic orientation appears in the XRD data.

If we compare XRD results before and after the 15-min immersion in aqua regia, it suggests that the treatment has eliminated GeO_2_ in the (103) plane [39], while promoting an etching along the (311) [40,41,42] direction (exposing this crystalline plane), to form the inverted pyramid structures.

Based on these data, the Scherer formula can evaluate the crystallites’ average diameter L in the direction perpendicular to the plane hkl, under the assumption that the line broadening is mainly due to the fragmentation of the crystal in small areas of coherent diffraction size *L* (<1000 Å) occurring during the chemical etching. This parameter *L* will be estimated using the following equation [43]:

(1) $$L\;\mathrm{hkl}\;=\;\frac{0.9\lambda }{b\cos\theta }$$

where *b* is the angular full-width at half-maximum (FWHM) of the diffraction peaks, θ is the Bragg angle defined by the peak position and λ is the source wavelength (K*_α_*_1,2_ of Copper = 1.5418 Å). The XRD data in Figure 6 allow calculation of the (311)-peak full-width at half-maximum (FWHM) for the etched samples of 1.0° ± 0.1°.

This calculation suggests that the crystallite size of the treated sample is 29.87 Å in the Ge (311) plane. In reality, this evolution is due to the growth of crystallites and can also be influenced slightly by experimental parameters such as local deformations induced by dislocations or variation of concentration in the case of solid solution [44].

Based on this analysis, the exposed facets in the inverse pyramid-shaped pits clearly seem to correspond to the (311) directions [45], and the diffraction peak evolution likely originates from concentration variations of the solid solution after etching in the perpendicular direction to the (311) plane [44].

### 2.4. Surface Chemistry Analysis Using FTIR Analysis

**Figure 7:**
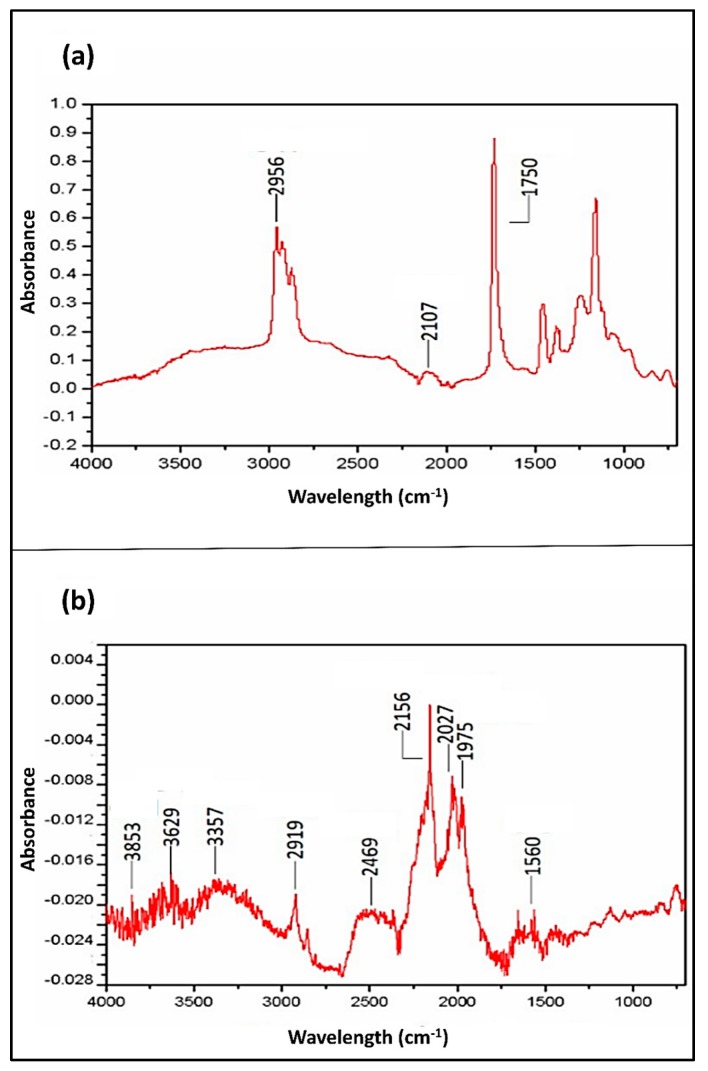
FTIR analysis of the Germanium sample. (**a**) Clean Germanium sample without any aqua regia treatment; (**b**) after a 15-min immersion in aqua regia at room-temperature.

For potential device integration, it is essential to know the surface chemistry for the nano-textured Ge. In this section, we investigate how the aqua regia treatment affects the chemical nature and the chemistry of the exposed surface. In the FTIR spectroscopy results of both etched and non-etched Germanium samples shown in Figure 7, the assignment of peaks was based on several literature sources and it is summarized in Table 3. Even though the same frequency vibration could be assigned to more than one atomic group, we notice the domination over four types of atomic bonds: (1) a large Ge–H*_x_* vibration between 1875 and 2250 cm^−1^; (2) O–H bonds which essentially appear between 3350 and 3855 cm^−1^; (3) Carbon based vibrations in several frequencies; and (4) N–H vibrations.

The most challenging ascription relates to the identification of the Ge–H*_x_* components of the etched sample. Based on the literature, the Ge–H vibration appears between 1950–1990 cm^−1^ [50], which is consistent with the peak found at 1975 cm^−1^. Meanwhile, the 2027 cm^−1^ peak is most likely due to Ge–H_2_ [51]. Due to the collision which may happen between the two H atoms during hydrogen desorption, the Ge–H_2_ is unstable and can be easily reduced to monohydride [54].

Also, despite the fact that the 2156 cm^−1^ peak could be attributed to Ge–H_3_ bonding vibration [55], this is still not favorable considering the reaction leads to the formation of H_2_ in addition to H_2_O. Indeed, the H_2_ formation should dominate during the reaction enabling the etching of the surface. However, Ge–H_3_ formation could be more favorable with more hydride in the reaction [51,56], if terminations activating GeH_4_ are H_3_, H_2_ and H [51] or when working on removing H_2_O_2_-grown Germanium oxides (GeO*_x_*, *x* = 1,2) using HF [56]. For all those reasons this peak is more likely to correspond to a C≡C bond and this suggestion will be supported later with XPS analysis.

As for the O–H peaks at 2469 cm^−1^ and between 3350 and 3855 cm^−1^, they originate from hydrogen desorption, which expects to be accompanied by oxidation or hydroxylation of the surface [43]. Albeit the Ge–O_2_ removal based on XRD analysis shown in Figure 6, the sub-oxide is more difficult to eliminate and the ≡GeOH can remain as a stable form of the surface species.

The carbon-binding vibrations suggest the existence of some C≡C, C–H, and C–O bonds. While some of the carbon could have an organic origin or could be due to the anterior characterizations, previous studies on the stabilization of Ge–H in the air have shown that it is often accompanied by hydrocarbon contamination of the surface [57].

Finally, for the etched sample, the two peaks at 1560 cm^−1^ and 3357 cm^−1^ could indicate the presence of an N–H bond. This is expected considering the fact of HNO_3_ use.

Now, if we compare the two FTIR spectra of Figure 7, it is interesting to focus on the main vibrations related to the hydride termination (starting from 1900 cm^−1^). In this region, bare Germanium reveals two peaks, at 2107 cm^−1^ and at 2956 cm^−1^, both related to carbon vibrations.

Hence, it is clear that, except the C–H peak observed at 2919 cm^−1^ and 2956 cm^−1^ for the etched and the bare sample respectively, the hydride termination observed on the etched sample appears largely as a result of the treatment. However, the C≡C peak observed at 2107 cm^−1^ and at 2156 cm^−1^ for the etched and bare sample respectively, indicate, not a contamination due to an exposure to the air, but rather a polymerized carbon. This may due to previous SEM characterization. After a relatively long period under electron beam, the surface of germanium can become carbonized.

### 2.5. Deeper Understand of the Surface Chemistry Using XPS Analysis

XPS analysis was performed to better understand, identify, and quantify the elements at the surface and their bonding. Since we used HCl and HNO_3_ to etch Germanium, Ge, O, H, Cl, and N compounds are expected. A lot of C is also expected as the surface exposed to air and it has undergone several characterizations before XPS analyzed. Two samples were analyzed: one pristine germanium (not etched) taken as a reference and one etched with Aqua regia for 15 min.

XPS analysis was first performed using both AlK*α* and MgK*α* sources as shown in Table 4, because some peaks are superposed. Indeed, the Auger electron peak of Ge is situated in the same binding energy range as chlorine when Mg is used, and thus not allowing to correctly identify the presence of chlorine. Similarly, the Auger electron peak is found in the oxygen binding energy range when Al is used, which does not allow a proper analysis of the oxygen peak. Since no chlorine was detected in two samples, high-resolution analysis were done only with MgK*α* radiation shown in Table 5.

In the overview analysis, as shown in Table 4, we find an important fluorine contamination on the pristine Germanium and a low zinc contamination in the 15 min-etched sample. Those contaminations come from the improper handling of samples during the cleaning and previous characterizations, and they will not be the subject of interpretation. There is also a high carbon contamination on the surface of the two samples. As we explained previously, this may due to exposure to ambient air and previous SEM characterization. After a relatively long period under the electron beam inside SEM, the surface becomes carbonized and burned. This result is in agreement with FTIR spectroscopy in which we identified the existence of C≡C peaks in both etched and non-etched samples.

High-resolution analysis of the reference sample (pristine Ge) shows that the germanium present in the surface is in Ge^0^ form (binding energy (BE) = 29.4 eV), non-oxidized, and in oxidized form GeO_2_ (BE = 33.0 eV). The analysis of the carbon peak of this same sample shows a narrower peak (FWHM = 1.3 eV) than normal (normally FWHM = 1.6 eV) found when it comes to simple carbon contamination from hydrocarbons in the air. A narrower peak indicates a well-organized compound such as a polymer or high-density graphite. Finally, oxygen peak analysis indicates that the latter is present in part in Germanium oxide and hydroxyl groups in the surface.

Analysis of the 15-min etched sample shows that there is no more GeO_2_ present on the surface of this sample and the most part of germanium is in the Ge^0^ form. A small proportion of Ge^1+^ and Ge^3+^ are also found respectively at BE = 30.2 eV and at BE = 32.2 eV. The presence of a small quantity of Ge^3+^ shows the presence of a germanium sub-oxide Ge*_x_*O*_y_* (*y*/*x* is about 3/2). The Ge^1+^ could similarly indicate a sub oxide Ge*_x_*O*_y_* with *y*/*x* = 1/2, but it could also indicate Ge–H bonds [58]. An important carbon contamination is also present in this sample. As for the reference sample, this carbon contamination may come from polymerized carbon during SEM analysis previously made on these samples. This, thus confirms the attribution of the 2156 cm^−1^ peak in the FTIR analysis to C≡C and not to Ge–H_3_. For the future, it would be required to do the surface analysis before SEM analysis.

In summary, the treatment using the mixture HCl:HNO_3_ can lead to three possible scenarios regarding the surface chemistry: (1) The treated surface can be Cl terminated (Ge–Cl), (2) the treated surface can be H terminated (Ge–H), and (3) the surface consists of free dangling bonds (no termination at all) and the samples, in this case, oxidize with air exposure.

As no chlorine (Cl) was detected, the Ge–Cl scenario can be readily dismissed. Moreover, the XRD and XPS results confirm that germanium dioxide (GeO_2_) is efficiently cleaned and eliminated from the surface of the etched sample. This leaves only the hydrogen passivation (Ge–H) which the existence was discussed and supported by FTIR and XPS results shown in Table 3 and Table 5.

Finally, the observation of oxygen peaks with BE = 531.3 eV and BE = 532 eV could equally indicate the probable presence of a small quantity of sub-oxide (Ge–O) or Ge–OH due to Hydroxyl desorption. This suggests either that Ge–O exists in the surface before the treatment and the surface was not fully hydrogenated after the treatment, either the adsorption of hydrogen in ambient air is accompanied with oxidation and hydroxylation, or both and this does not have adverse effect as the surface remains stable and does not oxidize during exposure to the air after treatment.

### 2.6. Broadband Reflectivity Measurements

Our main purpose consists in achieving controllable and uniform nano-texturing of Germanium at low costs is to reduce the intrinsically high reflectance of bulk Germanium to enhance the light coupling efficiency for future Ge-based optoelectronic devices such as photo-detectors and photovoltaic devices. Indeed, the inverted pyramid-structure will trap the incident light. This will be particularly useful for homo- or hetero- junction Ge-based optoelectronic devices since all the light is wanted to be absorbed at this level and photons should not transmit. To observe this improvement, we performed reflectance spectroscopy between 950–1700 nm as a function of the immersion time using a spectrophotometer equipped with a 60 mm integrating sphere. The choice of this specific wavelength regions is imposed by Ge bandgap which is equal to 0.67 eV at room temperature. As the band gap is inversely proportional to wavelength, this means that Ge is unable to absorb long wavelengths higher than $\lambda_{max}\;=\;\frac{hc}{E_{g}}$ = 1.85 μm. Thus, this makes it suitable for near-infrared based opto-electronic devices such as photodiodes and solar cells just like Silicon for visible range (0.4–0.7 μm).

**Figure 8:**
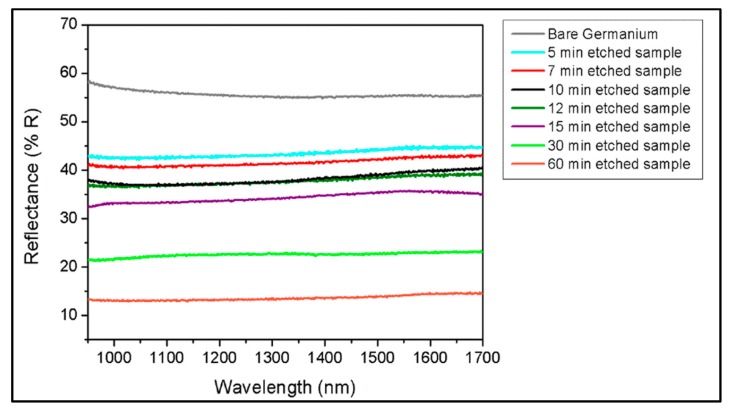
Broadband NIR reflectance of Germanium samples for increasing immersion times and comparison with a bare Ge sample.

As shown in Figure 8, the pristine Germanium reflectance varies between 54.8–58.5% between 950 and 1700 nm. A quick 5-min etching already yields a 12–14% decrease in reflectance. A long immersion further decreases the reflectance, which falls under 15% for a 60-min immersion. Based on previous sections, this corresponds to a mean depth around 2 µm and can reach almost 4 µm in peak-to-peak height. For opto-electronic devices integration, the junction is generally located close to the surface which make etching times between 5 and 15 min the region of interest as it induces to the formation of porous structures with mean depths between 0.31 and 0.75 microns. Further etching is still possible with longer etching times as we can note for 30 and 60 min’ baths.

**Figure 9:**
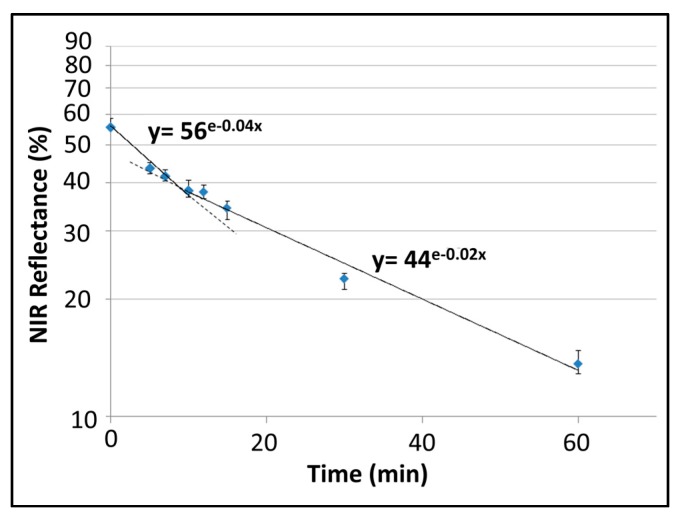
Average NIR reflectance (950–1700 nm) of Germanium samples for increasing immersion times and comparison with a pristine sample. The vertical bars show the minimum and maximum reflectance measured between 950 and 1700 nm for each etching time.

As such, this low-cost aqua regia treatment can decrease the total reflectance and generate large gain in absorbance from 950 to 1700 nm. Figure 9 clearly shows that the decrease in reflectance slows down as the etching rate saturates for longer immersion, largely due to the Germanium surface passivation with hydrogen and the weakening of the etching power over time.

## 3. Materials and Methods

The nano-texturing process is entirely realized in a fume hood under ambient laboratory conditions. All samples are produced using commercial Sb-doped n-type Ge (100) wafers with resistivity *ρ* = 0.1–0.5 Ω∙cm, which are purchased from MTI Corporation. These wafers are 2″ in diameter, 500 µm-thick and single-side polished (SSP). Nitric acid (HNO_3_) at 70%, hydrochloric (HCl) acid at 37% and Hydrofluoric (HF) acid at 48% concentrations, purchased from Sigma-Aldrich (St. Louis, MI, USA), while deionized water (DI-H_2_O) purchased from MAT laboratory.

First, the Ge wafers are cleaved into 1 cm^2^ pieces using a diamond scriber, then dipped for 1 min in 2% HF, rinsed with DI-water and blow-dried with ultra-high purity nitrogen (N_2_) gas. Then, cleaned samples are immersed immediately in a fresh aqua regia mixture (HCl:HNO_3_ at 3:1). This 3:1 ratio refers to the respective volumes of HNO_3_ and HCl solutions as received from Aldrich. After the desired etching time, the samples are removed from the solution bath and rinsed with DI-water, followed by N_2_ blow-drying. The nanostructured Ge samples are then sealed in clean closed containers for the different characterizations.

The morphological characterization is performed using a field-emission scanning electron microscope (FE-SEM, model Hitachi SU-70, Schaumburg, IL, USA), while the topography of the resulting surface structure is measured using an atomic-force microscope (Bruker, Billerica, MA, USA, model Multimode 8 equipped with ScanAsyst scanning mode) and analyzed using the Gwyddion freeware to extract statistics pertaining to the pit structure and the surface roughness. Unfortunately, it wasn’t possible to extract from the software the different values that the variable (height) can take from each iteration from pixel 1 to pixel *n*. So, it becomes not possible to calculate the standard variation around the mean height. However, it is important to precise that the mean height is calculated from the points defined by the pixels of the picture. For our measurements, we considered a picture of 512 by 512 pixels which results on *n* = 262.144 points. The mean height of 262.144 points is consequently very representative of the surface state. 

For potential optoelectronic device integration, it is essential to acquire a clear understanding of the surface properties following the nano-patterning process. To do so, crystallinity is analysed using theta-two-theta scan under X-ray diffraction (PANalytical X’Pert MRD 4-circle diffractometer, Amsterdam, The Netherlands), while atomic bonding on the surface is probed using FTIR (Thermo Scientific, Waltham, MA, USA, model Nicolet 6700 equipped with a Diamond Smart iTR Attenuated Total Reflectance module) and X-ray photoelectron spectroscopy (VG instrument, Waltham, MA, USA, model ESCALAB 3-MKII) equipped with an MgK*_α_* and an AlK*_α_* beam sources. 

Finally, the surface reflectance is measured using an NIR spectrophotometer (Perkin Elmer, Waltham, MA, USA, model Lambda 750) equipped with a 60 mm integrating sphere. The UV-VIS-NIR spectrophotometer setup consists of several blocks and compartments. First, we find the source system with its two deuterium and halogen lamp. Depending on UV, VIS, or NIR region, the appropriate source is selected. Then, the light passes through a monochromator which selects the wavelength depending on the selected range and the interval between two consecutive data. Once the monochromatic light is selected, this one should be free of noise. For this reason, the flux passes through a filter system to eliminate every kind of noise and to make the light smooth. At this level, the light will be directed to the chopper and will split to two identical quantities: One will be directed to incite a reference sample and the other one will be directed to incite the sample we want to characterize. Now, it is important to zoom-in the samples compartment. The samples are positioned at a tilt angle of 8° from the vertical. It is of course important to mention that the light coming from the source and directed toward the samples is oriented through different optical lenses and that the flux goes through a 60-mm integrating sphere before hitting the concerned surface. Samples are positioned on two different walls of this integrated sphere. Thereby, the reflected rays undergo different reflection inside the sphere in order to make uniform information, which will be read from the detector placed in the top of the integrated sphere. The detection system is constituted of a multiplier PMT for UV-VIS range and of a lead sulfide cell PBS for NIR range, which is the range of our interest. The use of an integrating sphere and a reference sample allow us to measure accurate relative percentages of reflectance.

## 4. Conclusions

Our results clearly show that the surface treatment using 3:1 aqua regia (HCl:HNO_3_) can allow a controllable, relatively uniform, and shallow nano-texturing of Germanium. Care should be taken to minimize the H_2_-bubbling, which can lead to a long-range second-order patterning of the surface for very short immersion times (under 15 min). Our results show that the anisotropic etching preserves the crystallinity while the etching occurs in a preferential direction along the Ge (311) planes, yielding inverted pyramid-shaped pit structures. Meanwhile, our results also suggest that the exposed Ge surface is hydrogen-passivated (Ge–H). This is particularly interesting as previous reports suggested that treatments using only HCl lead to Ge–Cl rather than Ge–H termination because of its higher bond energy [59,60]. Our experiments suggest that the addition of HNO_3_ in the mixture leads to the opposite result.

In summary, the aqua regia protocol provides a clean and dioxide-free nano-textured surface, which is also surface-stable and resistant to native oxidation due to hydrogen-passivation. The surface parameters’ evolution as a function of the immersion time shows that the process can relatively be well controlled to achieve the desired characteristics. Depending on the immersion (etching) time, the NIR reflectance can be reduced under 15% using a 2 µm-deep nanopatterning.

In the future, we believe this facile all solution-based surface treatment could be most useful to achieve low-reflectance across a broad spectrum to improve Ge-based optoelectronic devices.

## Figures and Tables

**Table 1:** Elemental analysis of the pristine untreated Germanium (Ge) (Section 1) and after a 60-min immersion in 70% HNO_3_ bath (Section 2), as seen in Figure 1d.

Mass Percent (%)			
Sections	C	O	Ge
Section 1	1.95	–	98.05
Section 2	–	22.90	77.10

**Table 2:** Surface statistics for different immersion times ranging between 5 and 60 min based on the AFM images from Figure 4 analyzed using the Gwyddion freeware.

Sample	Immersion Time (min)	Mean Height (µm)	Maximum Height (µm)	Roughness (µm)
(a)	5	0.31	0.70	0.06
(b)	7	0.33	0.76	0.06
(c)	10	0.35	0.81	0.07
(d)	12	0.42	1.02	0.07
(e)	15	0.75	1.37	0.13
(f)	30	1.27	2.16	0.19
(g)	60	1.98	3.28	0.40

**Table 3:** Attribution of infrared peaks to atomic bonds.

Valence Vibration Frequency (cm^−1^)	Attribution of Peaks to Atomic Bands	References
1560	N–H	[46]
1750	C=O	[46,47,48]
1975	Ge–H*_x_* (*x* = 1) or X=C=Y (X,Y)	[25,46,49,50,51,52]
2027	Ge–H*_x_* (*x* = 2) or X=C=Y	[25,46,49,51]
2107	C≡C	[46]
2156	Ge–H*_x_* (*x* = 3), X=C=Y or C≡C	[25,46,51]
2469	O–H	[46]
2919	C–H	[46,48]
2956	C–H	[46,48,49]
3357	N–H, O–H (H bonded) or O–H (carboxylic)	[46,48]
3629	Free O–H or C–H	[46,48]
3853	O–H (stretching absorption of COOH)	[53]

**Table 4:** XPS overview analysis using MgK*α* and AlK*α* sources on pristine and 15 min etched Germanium.

Element	Binding Energy (eV)	Relative Atomic %			
		Measured with a MgK*α* Source		Measured with a AlK*α* Source	
		Pristine	15 min Etch	Pristine	15 min Etch
**Ge**	31.9	5.4	3.0	7.6	5.1
**C**	285.1	74.6	86.6	75.1	83.2
**O**	532.1	12.6	10.2	13.3	11.2
**F**	689.8	7.3	–	3.9	–
**Zn**	1021.7	–	0.2	–	0.4

**Table 5:** XPS high resolution analysis to identify qualitatively single or composed elements of the surface.

Element	Binding Energy (eV)	Full Width Half Maximum (FWHM)	Identification	Relative Atomic %	
				Pristine	15 min Etch
**Ge**	29.4	1.4	Ge^0^	2.3	2.4
	30.2	2.0	Ge^1+^ (Ge–H or Ge*_x_*O*_y_*, *x* = 2, *y* = 1)	–	0.4
	32.2		Ge^3+^(Ge*_x_*O*_y_*, *x* = 2, *y* = 3)	–	0.5
	33.0		Ge^4+^ (GeO_2_)	3.3	–
**C**	285.0	1.3	C–C	80.7	81.8
	286.8	1.6	C–O	–	4.6
**O**	531.3	2.2	Ge–O, Ge–OH (hydroxyl in surface)	–	6.5
	532.0			13.7	–
	532.8		C–O	–	3.8
